# From Livable Communities to Livable Metropolis: Challenges for Urban Mobility in Lisbon Metropolitan Area (Portugal)

**DOI:** 10.3390/ijerph18073525

**Published:** 2021-03-29

**Authors:** Ana Louro, Nuno Marques da Costa, Eduarda Marques da Costa

**Affiliations:** Center for Geographical Studies, Institute of Geography and Spatial Planning, Universidade de Lisboa, 1600-276 Lisboa, Portugal; nunocosta@campus.ul.pt (N.M.d.C.); eduarda.costa@campus.ul.pt (E.M.d.C.)

**Keywords:** livable communities, livable metropolis, urban mobility, SUMP, Lisbon Metropolitan Area, indicators

## Abstract

Urban mobility plays an important role in addressing urban livability. The complexification and dispersion of travel due to the improvement of transport and the multiplication of our daily living places underline the relevance of multilevel territorial planning, recognizing that the knowledge of local differences is essential for more effective urban policies. This paper aims (1) to comprehend conceptually how urban mobility contributes to the urban livability from the local to metropolitan level and (2) to assess the previous relation toward a livable metropolis based on the readily available statistics for the Lisbon Metropolitan Area. Hence, a triangulation between conceptual, political/operative, and quantitative/monitoring approaches is required. The methodology follows four steps: (1) literature review focusing on the quantification of urban mobility within the urban livability approach; (2) data collection from the Portuguese statistics system; (3) data analysis and results, using principal component analysis (PCA) followed by cluster analysis (CA); (4) discussion and conclusions. In Portugal, although it is implicit, consistency is evident between the premises of recent urban mobility policies and respective planning instruments, such as the Sustainable Urban Mobility Plans (SUMP), and the premises of urban livability as an urban movement. Focusing on the national statistics system, the available indicators that meet our quality criteria are scarce and represent a reduced number of domains. Even so, they allow identifying intra-metropolitan differences in the Lisbon Metropolitan Area (LMA) that could support multilevel planning instruments. The results identified five principal components related to commuting at the local and intermunicipal level, including car use as well as social and environmental externalities, and they reorganized the 18 LMA municipalities into eight groups, clearly isolating Lisbon, the capital, from the others. The identification of sensitive territories and respective problems based on urban livability principles is fundamental for an effective urban planning from livable communities to livable metropolis.

## 1. Introduction

Urban mobility has been under debate in the last decades due to its important role in urban movements related to sustainable development, healthy cities, and livable cities, among others. On the one hand, urban mobility remains dynamic, due to the influence of the space–time relation and the constant increase of average speed, promoting access to a wider territory and more activities. On the other hand, it is linked to an unsustainable footprint related to a high dependence of fossil fuel, pollutant emissions, road accidents, excessive land use, and congestion [1]. 

Nowadays, territorial policies need to respond to the challenges of our daily lives that are more complex (in terms of either number of trips and destinations), which are reflected in the increasing of the multiplicity of living places. Hence, if, on the one hand, it is important to know in depth one territorial unit (e.g., municipality or parish), on the other hand, it is also important to strengthen the relational analysis between various territorial units to support more effective regional policies and enhance synergies to solve common urban problems or implement good practices [2,3]. 

The Mobility Package from the European Commission and Mobility Package—Territory, Accessibility and Mobility Management in Portugal (published by the Instituto de Mobilidade e Transportes Terrestres (IMTT)) promotes the national guidelines for mobility [2,3] and highlights the Sustainable Urban Mobility Plan (SUMP) as the key instrument for policies and strategies on urban mobility. The SUMP points to an integrative approach between people and territories and to a multilevel and transverse perspective to build more sustainable, healthy, inclusive, and livable places. This planning instrument presupposes the existence of a monitoring system to assess the progress of results and impacts generated by the respective action plan in domains such as accessibility, non-motorized modes, public transport, and environmental impact, among others. The planning objective of this instrument is consistent with the requisites for carbon neutral, livable, and healthy cities, as it intends to (1) interfere with the land use changes, reducing car dependency toward public and active transportation and encouraging the greening of cities, and (2) create a vision and involve citizens in the decisions, which promotes collaboration, leadership, and diverse investments, everything based on a systemic approach [4]. 

In parallel, the concept of urban livability is still under debate. As Balsas said, “Livable means many things to different people. It is a concept, like quality of life, that people seem to recognize, but is difficult to define in a manner that everyone understands. A livable place is safe, clean, beautiful, economically vital, affordable to a diverse population and efficiently administered, with functional infrastructure, interesting cultural activities and institutions, ample parks, effective public transportation, and broad opportunities for employment. It also connotes a sense of community” [5]. With the multiplicity of living places in larger areas, the concept of livability is transposed to the metropolis level as “a livable metropolis is the unique, functionally linked urban form that responds to the human needs, scale and aspirations and promotes the integral development of the person while improving its relationship with the space, both built and natural” [6] p. 42. The Chicago Metropolitan Agency is often referred as example of the livability approach at the regional level, considering that “livable communities are healthy, safe and walkable… offer transportation choices that provide timely access to schools, jobs, services, and basic needs… Are imbued with strenght and vitality, features which emerge from preserving the unique characteristics that give our diverse communities a “sense of place” [7] p. 37. In a broader level, the approach to a livable metropolis can be seen in two ways: (1) assuming the metropolis as a large community as an effect of scale, blurring the different realities, which is insufficient in the context of territorial planning; or (2) assuming the metropolis as a system of communities, considering the differences of each one, integrated by a transport system that guarantees the principles of integration, non-exclusion, reduction of environmental impacts, and offering employment, services, and commerce according to the different hierarchies of functions (recalling Christalerian principles) [8]. This second approach will support the paper. 

This paper has two objectives: (1) to comprehend conceptually how urban mobility contributes to the urban livability from the local to metropolitan level and (2) to assess the previous relation toward a livable metropolis based on the readily available statistics for the Lisbon Metropolitan Area. The interest of this work is the triangulation between the literature (conceptual approach), the guidelines for urban mobility policy instruments (political/operative approach) and the indicators that already exist in the Portuguese statistical system (quantitative/monitoring approach). In general, planning orientations promoted for urban mobility are implicitly consistent with the theoretical assumptions for urban livability. We identified a set of already existing indicators that represent the previous relations, albeit in a small number and covering a scarce number of domains. Recurring to some statistical analysis (principal component analysis and cluster analysis) based on the existing indicators already allows the identification of some relevant factors consistent with the literature (commuting at the local vs. intermunicipal level, car use, and social and environmental externalities), different profiles of municipalities (organized the 18 LMA municipalities into eight groups, clearly isolating Lisbon, the capital, from the others), and pointing out sensitive domains in certain municipalities, reinforcing the importance of reading them together toward a more livable metropolis, with utility for municipal and metropolitan mobility planning instruments.

This analysis could support planning instruments, especially Sustainable Urban Mobility Plans (SUMPs) at the municipal and metropolitan level, underlining the importance of evolving quality of life and urban livability concepts from conceptual discussion to operative policies. In addition, this could be useful to connect several municipal departments within a municipality council to promote multisectoral policies and maximize the impacts of the implemented measures, considering livable conditions as a main goal. This shows the relevance of approaching the academy and public institutions to advance in multisectoral and multilevel knowledge to support decision-making.

## 2. Literature Review 

### 2.1. Urban Mobility against Expected Urban Livability

In recent decades, the concept of sustainable development has become an umbrella of several policies in Europe and around the world, specifically regarding transport and urban mobility [2]. In parallel, the concept of urban livability has arisen, partly overlapping the sustainable development one. However, a distinctive aspect should be highlighted: “Sustainability is concerned with achieving long-term goals of economic, environmental, and societal wellness, primarily regarding future demands of continuous growth. Livability, on the other hand, is concerned with currently available conditions of a place to achieve immediate effects in meeting the urban needs of the residing community” [9].

Two key elements of urban livability must be considered [3]: (1) the urban environment, highlighting the symbiosis of urban form, the expression of the physical environment, and urban functions, reflecting potential and real uses of that environment (at the city level); and (2) individual preferences, considering people’s needs and personal values (at the individual level). It is in the interrelation of these two key elements that urban mobility arises, as this domain is related to many aspects of livability: “Human needs and available urban functions define the destination that a person intends to reach, while personal values might affect the transportation mode. Moreover, urban form influences the actual route according to the infrastructure. Another aspect of the relevance of mobility is the ability to represent effective access to urban functions” [3].

In this sense, urban mobility could be considered as one of the “ingredients” of livable cities; it promotes planning principles for mobility from a multilevel perspective, from pedestrian areas to international rail systems, with a commitment to social equity and a sustainable environment [10]. This is reflected in some urban movements, such as smart growth, that present a holistic perspective of cities and their communities through mixed-use development, higher urban density, and efficient infrastructure to get more dynamic communities at the social and economic level, proposals to slow down traffic, better levels of road safety, and privileging pedestrians and cyclists over cars [11]. 

There are several urban mobility problems that interfere and cause negative consequences regarding the expected high levels of quality of life and livability.

Individual motorized transport is increasingly used to the detriment of public transport and active or soft modes. There has been an increased and excessive level of motorization in the last decades in urban and metropolitan areas, which is supported by the use of individual transport, generating problems such as congestion, road accidents, excessive fuel consumption, and pollutant emissions. This leads to consequences such as infrastructure cost, time cost, behavioral and psychological disorders, and a lack of physical activity and social interaction. On the other hand, active transport such as walking and cycling could improve physical activities, minimizing health issues such as cardiovascular and respiratory diseases, diabetes, obesity, and mental disorders, among others [12,13,14,15,16].

As is reinforced in sustainable, livable, and healthy urban movements, travel for commuting or to reach goods and services reflects, in part, the condition of the territory, specifically the availability of sufficient and diverse jobs, shops, services, and leisure areas nearby residences [17]. In a sustainable urban approach, travel should be as short, quick, and cheap as possible, revealing the ability of a place to serve the needs of the community [18]. Frequently, high levels of walkability are related to better livable areas, as walkability considers the comfort, convenience, and safety conditions to walk [19]. Commuting is widely studied due to the high regularity, reflecting economic and labor dynamics at the local, municipal, and regional level and land use [20]. Thus, the obligation to travel longer distances causes several problems, specifically a greater need for motorized transport, with consequent pollutant emissions and fuel consumption, with a negative impact on health, greater time consumption, financial constraints for families with lower incomes, and difficulties for people with reduced mobility when the transport system is not adapted to everyone, which is one cause of social exclusion [21]. 

Another component is time spent, which is strongly related to the domains of transport mode and travel destination. Here, long distances to workplaces or other destinations along with less rapid or less flexible modes, or traffic congestion, may increase travel time. This may represent a mismatch between people’s needs and the urban conditions of a community or metropolis. Some people’s personal choices when looking for goods, services, or places of leisure may require longer travel, generating excess travel if they cannot satisfy their needs near home or the workplace. The excessive time consumed in traveling, especially when focused on daily commuting, is time lost for the possible accomplishment of other activities or to rest, causing problems with stress and exhaustion [22,23,24,25]. 

The level of accessibility is an important aspect for an efficient polycentric urban model, which is considered one of the most urban sustainable models. Here, we can consider accessibility to a transport network or some transport mode according an “availability” perspective. Lower levels of accessibility contribute to situations of social exclusion [2]. 

Affordability is another one of the criteria related to the social exclusion, especially focusing on the needs of the most vulnerable groups, such as older people, families with low income, or people living in areas of transport scarcity. Financial limitations preventing the use of transport, private or public, could bring circumstances of greater difficulty in accessing health equipment, education, or fundamental goods and services, reinforcing the situation of social exclusion. This issue is commonly supported by local and national governments in cooperation with transport operators, with subsidiarity emerging as a tool to make the cost for users more affordable [25,26,27]. 

Road accidents are among the most negative externalities of transport, as the World Health Organization (WHO) estimates 1.2 million road deaths per year. In fact, Black notes that a transport system that kills its users can never be sustainable. Beyond the physical and psychological damage to victims which, in the extreme, can lead to death (with pedestrians and cyclists being more vulnerable), there are other economic costs of damage, insurance, and medical care [28,29,30,31]. Road accidents are influenced by urban density and the road infrastructure, but also the individual behavior of users of the transport system (as drivers, pedestrians, cyclists) [32]. 

Driving behavior is influenced by the conditions of the road infrastructure, the built environment, or the weather, but also by individual factors, including the driver’s status (driver’s license, level of alcohol intake, influence of illegal substances, level of tiredness) or even the attitudes toward driving, which are associated with compliance with safety and speed rules, and risky driving [33,34,35,36].

Focusing on the environment, transport is seen as a great source of pollution in the form of atmospheric pollution, noise, and vibration, which is mainly related to motorization, provoking respiratory, cardiovascular, and neurological diseases and subsequently contributing a great deal to the mortality rate [37,38,39,40]. Transport also promotes global warming due to the emission of greenhouse gases [41], which in turn also has an impact on health, especially in extreme weather events (heat, cold, rain, fog) causing negative impacts, especially affecting more sensitive groups such as children and the elderly [42,43,44]. 

Lastly, fuel consumption is one of the most studied issues nowadays, considering that reducing it is one of the main goals of sustainable development, in order to reduce the dependence on non-renewable fossil energy resources and combat some effects of climate change [31,45]. The transportation sector represents the higher final energy consumption, mainly due to road transport, in Portugal (37%) than in the EU-28 (31%), despite efforts to minimize total energy consumption [46,47]. This could be minimized through the reduction of motorized travels (number and length) replaced by online trading or telework [48], or vehicles technologically and energetically more efficient and less polluting [49,50]. 

### 2.2. Measuring Urban Mobility as a Component of Urban Livability

Urban livability is considered as a multidimensional concept composed of various criteria and subcriteria [51], which is often associated with rankings and indices, such as the Global Liveability Index (GLI) [9], the Economist Intelligence Unit (EIU) Liveability Ranking, the Mercer Quality of Living Survey, and the Organization for Economic Co-operation and Development (OECD) Better Life Index. However, some limitations are identified, as this approach “does not consider the person–environment relationship at different thematic and spatial scales, especially in a transferable way” [3]. Hence, the unavailability of data for all territorial units does not allow for replication, and the exclusive focus on cities or regions does not allow for an analysis from a multilevel perspective, which hide intra-urban or intra-regional differences, which are relevant aspects in the context of policy-making [51]. 

In a systematic study that included 67 articles, Khorrami and colleagues [51] recognized five main domains to measure “urban livability”:

(1) Economic vibrancy and competitive economic performance, economic openness, and infrastructure.
(2) Environmental friendliness and sustainability: pollution, depletion of natural resources, and environmental initiatives.
(3) Domestic security and stability: crime rate, threats to national stability, and civil unrest.
(4) Sociocultural conditions: medical and health care, education and housing, sanitation and transportation, income equality and demographic burden, and diversity and community cohesion.
(5) Political governance: policy-making and implementation, government system, transparency and accountability, corruption.

Within those, urban mobility topics or indicators cover the following: (1) active transport and public transport, (2) transport infrastructure, (3) accessibility, (4) road safety, (5) environment, and (6) economy and energy (Table 1).

## 3. Methodology

Several methodologies are used to quantify the urban livability, such as qualitative Delphi methods, analytical hierarchy process (as the Technique for Order Preference by Similarity for Ideal Solution (TOPSIS) and entropy), cluster analysis, factor analysis, principle component analysis, geographic information system (GIS) and spatial modeling, Economist Intelligence Unit and Mercer city rankings, comprehensive marking or standard method, livable level integrated index, neural networks, and The Global Liveable Cities Index (GLCI) [51]. While some of these methods allow the cartography of several indicators, other methods allow us to analyze the information in a correlated way, understanding which indicators exert greater influence in each area (e.g., factor analysis) or how the territorial units under study can be grouped according to their similarities (e.g., cluster analysis). There is a wide variety of indicators regarding urban mobility in the context of urban livability. Some indicators are fully available from official statistical sources, others are produced using surveys or interviews, others are collected using specific tools (e.g., sensors for measuring noise or air pollution), still others are produced using geographic information systems.

### 3.1. Methodological Steps

**Figure 1:**
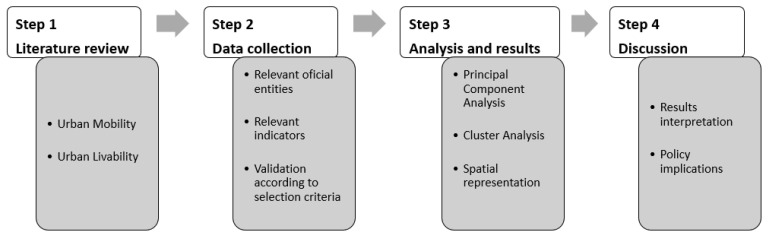
Methodological steps.

The methodology followed four main steps (Figure 1). 

The first step concerns the literature review about the relation between urban mobility and urban livability and what measures could represent it, as the utility of this approach in the context of SUMP. The second step is focused on data collection, considering, on the one hand, the geographic context and territorial level, in this case the Lisbon Metropolitan Area and its municipalities, and, on the other hand, readily available data from official sources, such as Statistics Portugal, the National Road Safety Authority, the Portuguese Environment Agency, and the Directorate-General for Energy and Geology, among others. The criteria for selecting indicators are also present. The third step is related to the statistical analysis. The constructed dataset was subjected to principal component analysis (PCA), which is a statistical procedure to reduce the number of variables to a smaller number of dimensions. The results from PCA made it possible to proceed with cluster analysis (CA), to group similar municipalities within the LMA. Both procedures allowed us to understand the diversity of municipal reality within the LMA about this subject. Geographic information systems were used to map the obtained data. Lastly, the fourth step is to discuss the results from statistical and spatial analyses.

### 3.2. Data Collection and Main Sources

**Figure 2:**
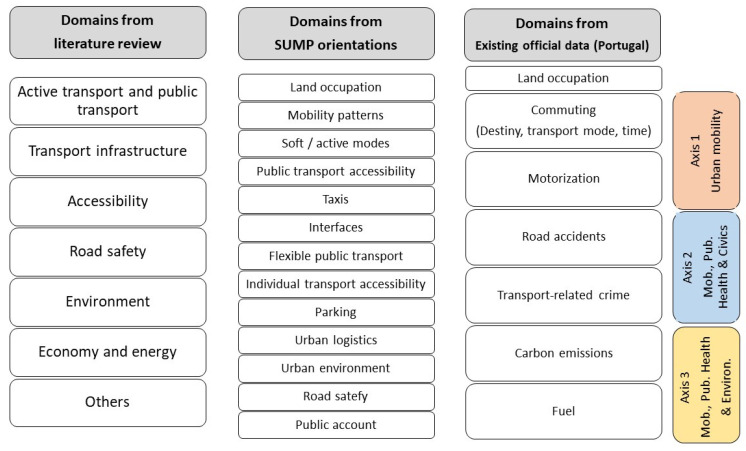
Domains of urban mobility (literature review, Sustainable Urban Mobility Plans (SUMP) orientations, official data in Portugal).

This research is intended to be useful for public entities working on urban mobility and sustainable, healthy, and livable places with a multisectoral approach. Hence, only readily available data from official sources were selected. The selection of statistical information should satisfy several criteria: (1) it must be produced by official entities and be fully available, free of charge and online, without any access restrictions, thus fulfilling the quality and uniformity criteria of retrieval and treatment; (2) it must be actualized regularly, recent, and available for at least two years (therefore, indicators displayed for only a short period and/or originating from studies or research projects were excluded); (3) it must exist for all municipalities in the Lisbon Metropolitan Area (with rare exceptions); and (4) it must be consistent with the domains that emerged from the literature review (Figure 2). 

Given these criteria, 17 indicators were considered in this study covering subjects such as commuting (main destination, main transport mode, time spent), motorization, road accidents, transport-related crime, carbon emissions, and fuel consumption, along with population density as a supportive variable. Table 2 presents the metadata of those indicators (unit, recent year of publication, source).

### 3.3. Statistical Analysis

Two statistical procedures were applied using the SPSS statistical package (SPSS Statistics for Windows, version 25.0, IBM Corporation, Armonk, NY, USA). 

First, principal component analysis (PCA) was applied, with the set of 17 indicators in Table 2 at the municipal level as input data, aiming to identify a smaller number of components reflecting the combined mobility dynamics and respective social and environmental externalities. In this study, we considered the following PCA outputs: descriptive statistics table (mean, standard deviation, etc.), correlation matrix, and the loadings and component eigenvalues. Then, the obtained scores for each factor were mapped, allowing spatial visualization of the PCA results.

PCA “is a technique for reducing the dimensionality of such datasets, increasing interpretability but at the same time minimizing information loss” [52]; in other words, this statistical procedure transforms the original variables into a new, smaller range of variables named principal components, and the first principal component is “the linear combination of observed variables that maximally separate subjects by maximizing the variance of their component scores” [53]. The second component emerges from the residual correlations. This and the subsequent components “extract maximum variability from the residual correlations and are independent from all the other components” [53]. It is assumed that “the extracted components represent most of the variance of the original data set and can be used in further analysis” [53], so PCA can be represented mathematically as

PC_1_ = a_11_X_1_ + a_12_X_2_ + … + a_1n_X_n_

…(1)

PC_m_ = a_m1_X_1_ + a_m2_X_2_ + … + a_mn_X_n_,

where PC_1_ to PC_m_ represent the extracted principal components, X_1_ to X_m_ are the set of variables, and a_mn_ represents the weight of the *m*th principal component and *n*th variable. The eigenvector of the correlation matrix represents the weight of each principal component, and the eigenvalue is the variance of each principal component.

Cluster analysis (CA) was the second statistical procedure used in this research. In this study, cluster analysis allowed the identification of groups of municipalities that share common characteristics, in this case based on the outputs of CA. Cluster matrix data were also mapped using ArcGIS, in order to facilitate a territorial representation of results.

CA is a statistical technique that allows the classification of several units in groups, named clusters, that are relatively homogeneous and heterogeneous among groups, based on a set of characteristics as variables [54]. The classification method chosen was hierarchical cluster in an agglomerative approach (from *n* clusters equal to the number of cases, to get to 1 cluster), creating subclusters within a cluster or, in other words, a sequence of partitioned clustering [55,56]. This choice could have some problems, such as the use of different measures with different weights and the risk of double counting related to the correlations between variables. Therefore, the data extracted from principal component analysis were used to minimize the limitations. 

## 4. Lisbon Metropolitan Area as Study Area

**Figure 3:**
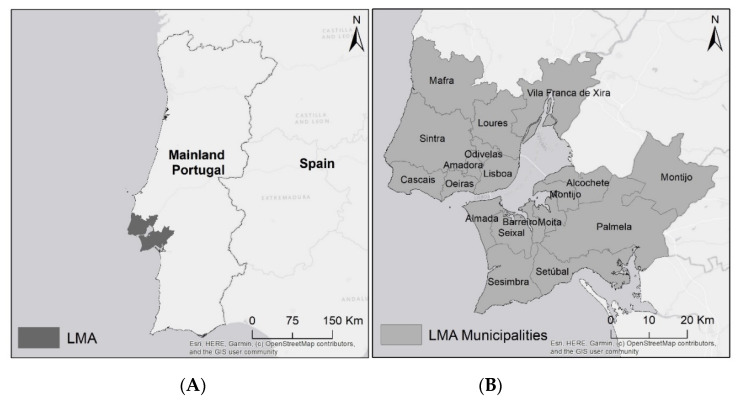
(**A**) Lisbon Metropolitan Area and (**B**) its municipalities.

This paper focuses on the Lisbon Metropolitan Area in Portugal. LMA is composed of 18 municipalities (Figure 3), with Lisbon city having the greatest relevance. With 2,846,332 inhabitants in an area of 3015 km^2^ and a population density of 944 inhabitants/km^2^, LMA is the second most populated region in Portugal, but it has the highest population density by far. Around 96% of residents live in “predominantly urban areas” [57]. LMA has seen considerable transformations over the last decades (1981–2011): 14% rise in population growth (from 2,482,276 to 2,821,699), 42% rise in families (from 810,770 to 1,147,775), 45% rise in buildings (from 308,814 to 448,957), 75% rise in dwellings (from 852,834 to 1,487,858) [58], and 39% increase in the urban area [59]. On the other hand, there was a decrease in the number of establishments (−13%) and employed persons in establishments (−5%) (2003–2013) [58].

During the last decades, LMA experienced an urban sprawl phenomenon result from the residential mobility and deconcentration of economic activities [54]. Lisbon city and the adjacent area were and still are the densest areas within LMA, while the most distant areas of the city are the least dense. However, Lisbon is continuously losing inhabitants who move to adjacent areas or near main roads in peripheral areas (where the transport network is suitable for general needs and the housing prices are more accessible), and more recently to new periurban and urban areas.

This metropolitan area is divided by the Tagus River, which is a natural factor that has always conditioned the evolution of urban settlements and transport networks. Hence, the two banks present significantly different urban occupation. About three-quarters of the residents and buildings and two-thirds of households are concentrated on the north side, although the highest growth rates between 1981 and 2011 were recorded on the south side. Between 1981 and 2011, Lisbon city registered a population drop by about 32%, from 807,937 to 547,733 inhabitants, while suburban and periurban areas increased their population and urban density [58].

## 5. Results

This section addresses the results of principal component analysis and cluster analysis based on the 17 indicators described in Table 1.

### 5.1. Results of PCA

PCA identified five components, with an explanation of total variance of 86.7%. Loadings and component eigenvalues are shown in Table 3.

**Figure 4:**
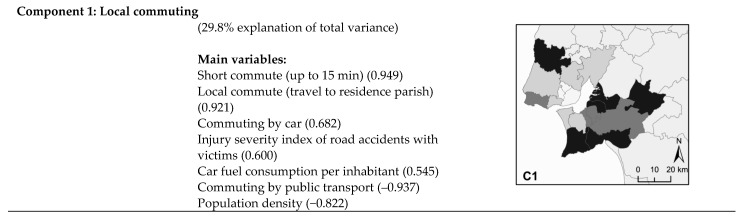

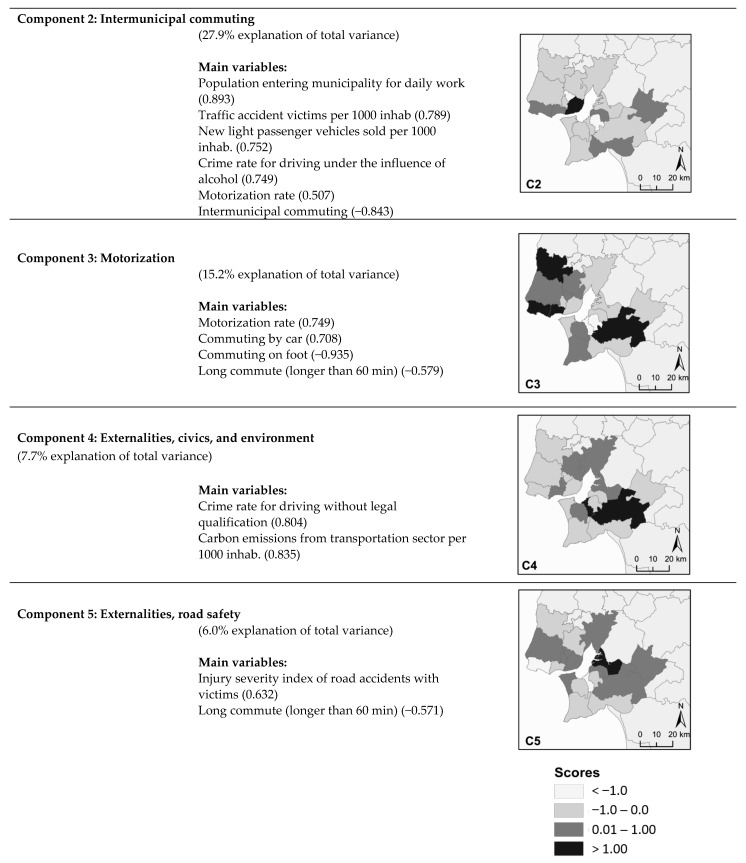
Principal component analysis (PCA) components, explanation of total variance, main variables, and spatial representation of each component in Lisbon Metropolitan Area (LMA) municipalities.

This interpretation is based on component loadings that represent the correlation coefficients between original variables and generated components. Each variable is present in all components but with different weights, allowing one variable to be part of the explanation of one or more components at the same time. The explanation of each component is defined by the variables with higher levels of correlation. In this study, we considered the most explicative variables to be the ones with correlation between 0.5 and 1 (moderate and strong positive correlation) and between −0.5 and −1 (moderate and strong negative correlation). Based on the scores of each component in every municipality, it was possible to generate a spatial representation for each component (Figure 4).

Regarding the first component, Mafra on the northern bank and Alcochete, Montijo, Setúbal, and Sesimbra on the southern stand out here (high positive score). They combine short and local commutes with the individual motorized option in low-density areas, compared to Lisbon, Amadora, Odivelas, and Loures (high negative scores) on the northern side, which have high-density areas and greater use of public transport.

The second component combines commuting by car and negative social externalities related to road accidents and crime (driving without a license or under the influence of alcohol). Lisbon has the highest positive score, followed by Setúbal, Oeiras, Montijo, and Cascais, in contrast to most of the LMA municipalities, especially Odivelas and Moita.

The third component includes municipalities with negative scores such as Barreiro, Moita, Alcochete, Almada, and Setúbal, mainly on the southern bank, with less car use in favor of pedestrian mode for short and medium commutes, and more positive scores for Loures, Palmela, Cascais, Mafra, and Oeiras, clearly reflecting that the car is the main commuting option.

For the fourth component, the municipalities of Palmela, Barreiro, Loures, and Seixal have the highest positive scores, with behaviors less consistent with expectations for healthy municipalities (high crime rate related to driving and high levels of carbon emissions from transport), differing from Almada, Cascais, Sintra, Sesimbra, and Mafra, with the highest negative scores.

Finally, the fifth component includes Alcochete, Odivelas, Almada, Vila Franca de Xira, and Sintra, with the highest positive scores, and Barreiro, Cascais, Moita, and Sesimbra, with the highest negative scores. This is related to a positive score on the injury severity index of road accidents with victims and a negative score for long commutes.

### 5.2. Cluster Analysis Results

**Figure 5:**
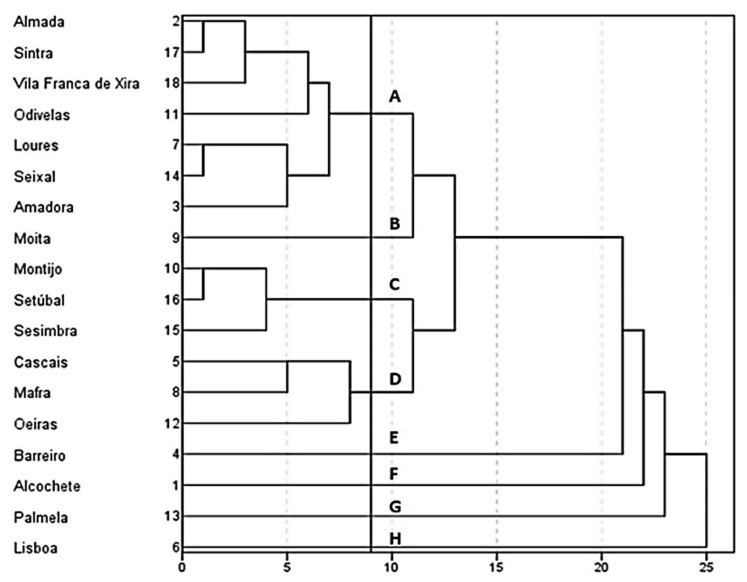
Cluster analysis dendrogram (cut-off line at distance 9).

**Figure 6:**
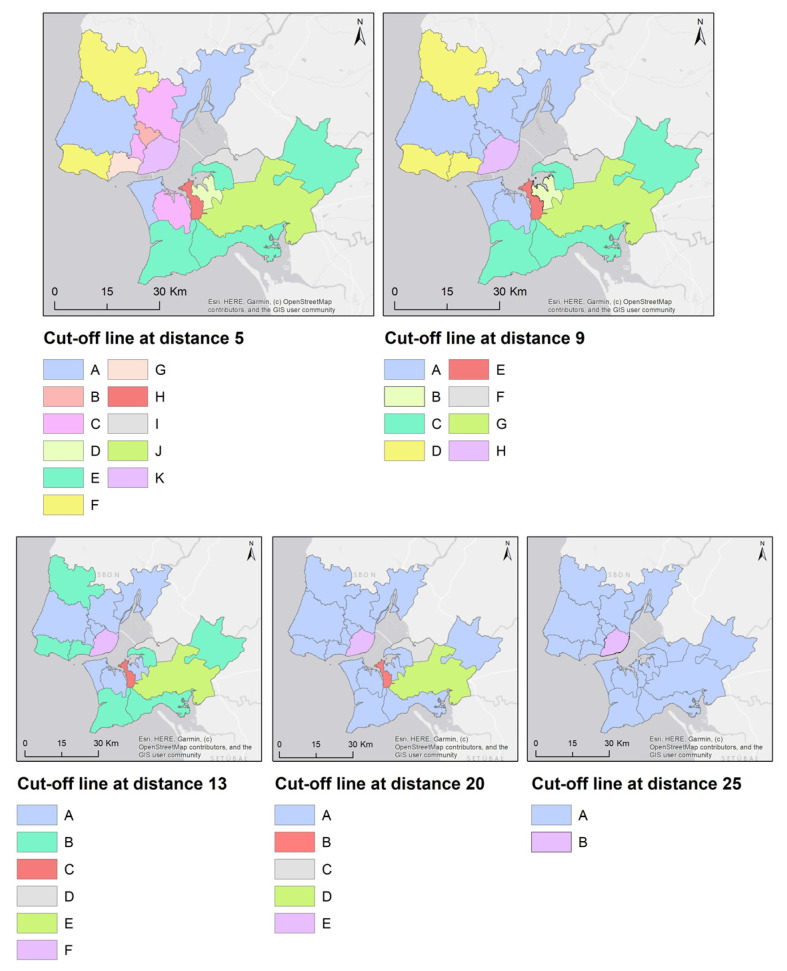
Spatial pattern based on cluster analysis (cut-off lines at distances 5, 9, 13, 20, and 25).

Figure 5 shows a dendrogram of the hierarchical classification of the cluster analysis, and Figure 6 shows the spatial pattern of the clusters considering diverse cut-off lines. 

For more specific analysis, the spatial pattern from the cluster analysis was used considering the cut-off line at distance 9.

Group A includes Almada, Sintra, Vila Franca de Xira, Odivelas, Loures, Seixal, and Amadora. It is characterized by a negative score on components 1 and 2, with a smaller proportion of short and local commutes, a large proportion of commuting to other municipalities, and a low level of attraction of employees, as reflected by the number of people who travel to work daily. 

Group B only includes Moita, with high negative scores on components 2 and 3, with the highest proportion of pedestrian commuting, the lowest number of new vehicles sold per 1000 inhabitants, a low level of car use, and a low motorization rate. It also has a high proportion of long commutes, and commuting to other municipalities, and a lower attraction of employees as reflected by the low number of people entering the municipality to work. 

Group C includes Montijo, Sesimbra, and Setúbal, with a high positive score on component 1, the highest proportion of local commuting, and the second highest proportion of short commutes, along with a high level of car use and a high injury severity index of road accidents. 

Group D brings together Cascais, Mafra, and Oeiras, with a high positive score on component 3, which was reflected in more commuting by car and a higher motorization rate, a lower proportion of pedestrian commuting, and the second lowest proportion of long commutes (above 60 min). 

Group E represents Barreiro, with a very high negative score on component 3, with less car use for commuting and a lower motorization rate, in contrast to a high positive score on component 4, due to the high crime rate based on the lack of a legal driving license. 

Group F only includes Alcochete, with a high positive score on component 1, due to the high proportions of pedestrian and car commuting, and a very high positive score on component 5, due to the severity of road accidents. 

Group G highlights Palmela, with a high positive score on component 3 due to the high motorization rate, and a very high positive score on component 4, with the highest volume of CO_2_ emissions per 1000 inhabitants and the highest crime rate for driving without a legal license. 

Finally, group H is made up of only Lisbon, reflecting the great discrepancy between the city capital and the other municipalities of LMA. It has a high negative score on component 1, as a result of having the lowest proportion of local and short commutes and the highest proportion of commuting by public transport. The high ratio of road accident victims per 1000 inhabitants is contradictory to the low injury severity index of road accidents, which is justified by the lower speeds of urban traffic. It also has a very high positive score on component 2 due to the highly polarizing function of employment, which is reflected in the lowest level of commuting to another municipality and large number of non-resident workers who travel to the city daily. The role of the car is evident in the high motorization rate and high ratio of new vehicles sold per 1000 per inhabitants.

**Figure 7:**
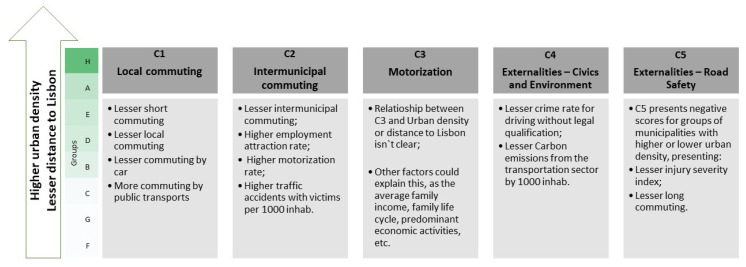
Relation between PCA components and clusters according to urban density and distance to Lisbon.

This reflects some important phenomena when we analyze the results obtained from PCA and CA and relate them to other variables such as urban density, distance to Lisbon, and employment attraction rate (Figure 7 and Appendix A). The results illustrate the relation of urban mobility, which is framed by the urban livability concept, with territorial urban development, considering that in areas with higher urban density (closely related to the distance to Lisbon) and local commuting (in the residence parish and taking up to 15 min), intermunicipal commuting is less evident, which is partly justified by the higher rate of job attraction. Public transport is more relevant as the commuting mode, which is supported by the better conditions of available services, despite the higher motorization rate. Regarding the social and environmental externalities, Lisbon and its surroundings demonstrate a lower crime rate associated with a lack of driving qualifications, lower injury severity index, and lower carbon emission ratio from transportation.

## 6. Discussion

The objective of this paper was to comprehend conceptually how urban mobility contributes to the urban livability from the local to metropolitan level and to assess the previous relation toward a livable metropolis based on the readily available statistics for the Lisbon Metropolitan Area. 

PCA generated five components: two strongly related to commuting at local and intermunicipal levels, one associated to the importance of the automobile, and the last two factors related to environment and social externalities. Based on PCA factors, cluster analysis showed diverse realities in the Lisbon metropolitan context and identified the great isolation of Lisbon, the capital; there was a certain homogeneity on the north bank, separating municipalities adjacent to Lisbon that have a great economic relationship with it, from more autonomous and/or peripheral municipalities; and there was a greater variety in the southern bank with five distinct groups: one associated with Lisbon, one integrating the most peripheral municipalities, and several isolated municipalities on the Tagus riverside arch. 

First, and despite the decentralization of employment, housing, and activities in recent decades, the data illustrate how the commuting model is still very centered in Lisbon. The dynamics related to the evolution of the road network led the complexification of origin–destination commuting, especially given the increased volume of travel between peripheral municipalities by car [17,60]. This pattern reflected the negative externality of the dispersed urbanization model that characterizes these territories (such as Mafra, Alcochete, Palmela, and Montijo) [61,62,63]. On the other hand, municipalities with greater density (Lisbon and the surroundings) were also those with employment attractiveness, which generated more intermunicipal travel and longer commutes based on individual transport in order to reduce time and distance. More intra-parish and/or short-term trips are more evident in less dense municipalities, as an answer to the local economic environment, with the use of active modes contributing to healthier and more livable communities [17,64].

The second aspect to be mentioned is that the use of public transport had a strong relationship with a higher population density, which is frequently related to more consolidated territories where the provision of public transport was more consistent [65,66]. It has been suggested that a higher population density can be a determinant of a greater mix of uses and functions in the territory, allowing better access to goods, equipment, and services, generating territories with opportunities, which has a positive impact on the development of livable territories [8]. This highlight the importance of reading the territory, the various transport infrastructures, other service networks (e.g., health, education) and the characteristics of the communities together. For example, other studies showed that it was in city centers that the pattern of the collective and active transport network was most favorable, while in suburban areas, there was a greater need to own a car to meet the needs of families. Particularly, in suburban areas with poor public transport networks, families unable to own a car tend to be excluded from social and economic participation, generating cycles of poverty [67]. 

The third aspect was focused on road accidents and air pollution, two of the main negative externalities that result from the greater use of individual transport and longer distances, which was reinforced by the inability of public transport in peripheral municipalities. These negative externalities contributing to the negative sense of a livable urban organization at all levels [8,68,69]. Although pedestrians and cyclists are the most sensitive elements in the global context of the transport system, studies have verified benefits in the modal shift from motorized to active transport, with more benefits than risks for all users [70,71].

These results were consistent and complementary to other PCA applied to the same study area, considering a table with sociodemographic and housing indicators together with commuting data at the parish level [54,65]. For example, Marques da Costa [72] identified four major territory typologies strongly related to commuting: (1) close occupations and short commutes in Lisbon; (2) close occupations and long commutes in the immediate outskirts of Lisbon and the railway axes of Sintra, Cascais, and Vila Franca de Xira; (3) dispersed occupations and external dependence on employment and longer commutes in Oeiras, Cascais, Loures, Vila Franca de Xira, Sesimbra, Palmela, and municipalities near the Tagus river (south bank); and (4) dispersed occupations and less dependence on external employment and short commutes in Montijo, Alcochete, Palmela, V. F. de Xira, Mafra, and Sintra. The patterns found in this analysis showed a strong relation with sociodemographic characteristics (age, education) and economic dynamics (employment, unemployment) [54]. 

The relevance of this study is the triangulation of the conceptual, the political/operative, and the quantitative/monitoring approaches of the discussion about urban mobility for urban livability, considering the already existent statistics for Lisbon Metropolitan Area, Portugal, allowing to differentiate the diverse realities within the metropolitan area. Despite the scarcity of indicators about urban mobility, the existent ones already represent the main axes of the relation urban mobility—urban livability (e.g., commuting, as a relevant regular travel, environment and health/social indicators) and allows us, through multivariate analysis, to identify several municipal profiles according their similarities. Hence, it is imperative to enhance urban livability, and the issues of urban mobility must be considered in a multiscale approach, from the local to the metropolitan, allowing not only livable communities but also livable metropolises, since the latter is the scale of experience of a considerable number of families, not only for work but also to satisfy all other needs (as in LMA, where 40% of workers and students need to get to other municipalities to work or study [58]).

However, several limitations should be highlighted. The first is the small number of available indicators related to the actual data of the national statistics system. In addition, the study included only indicators resulting from the global influence of all factors, especially impact indicators. It should be noted that there were no indicators associated with affordability, as a national measure was recently implemented regarding the price of public transport passes, creating a pass for one price that covers transport between all LMA municipalities. Indicators related to accessibility levels were also not included, despite a variety of these indicators being prepared by Statistics Portugal.

Second, there are statistical limitations involving innovative domains of urban mobility that could reinforce the approach to urban livability related to (1) the total lack of data, (2) the lack of frequent updating of data, (3) the lack of definition of data quality criteria for all producers, (4) the lack of interoperability between data producers and between producers and statistical platforms, and (5) the unavailability of existing data for civil society. This includes domains such as vehicle sharing perception and use [73,74]; perception of the quality of the transport system and the potential for modal transfers [31,60,75,76]; public transport system conditions, such as network coverage and frequency [65]; the use of new electric vehicles or more efficient vehicles [65,77]; societal and environmental externality costs [72,78,79,80]; and levels of accessibility to health, education, culture, and leisure equipment, and/or services [81,82], which are representative indicators of social inclusion of people with reduced mobility [83]. 

Other domains should be considered as context indicators due to their strong influence: sociodemographic, employment, and economic activity indicators, and others related to the physical environment, urban model, and land occupation [84,85,86].

One last limitation was related to the territorial level. In this case, the indicators under study were applied at the municipal level, which, in aggregate, allows us to obtain an image of the LMA. However, this option hides relevant intra-municipal differences [87]. On the other hand, some impacts exist only at the metropolitan scale, which diminishes their usefulness for local or municipal policies and planning instruments. 

Such limitations can be minimized by using other tools to collect data regularly, such as the information from surveys [3], transportation ticketing systems, GPS or smartphones of individual vehicles [86,88], and the generation of new indicators using other methodologies, such as multicriteria analysis, geographic modeling, spatial analysis, scenarization, and PCA considering context indicators, other scales, or a large number of territorial characteristics [54,64,82]. 

It should be noted that LMA is already developing a shared information and decision system [89], which includes, among others, health, transport, and energy working domains [89], making possible a better interpretation of the contribution of urban mobility to urban livability at several levels in the case of strengthening the indicator system. Hence, further research could support (1) the ongoing system; (2) the Lisbon Metropolitan Area (entity) as the Transport Authority to LMA, which has competencies for defining the strategic objectives of the mobility system, planning, and operating the public passenger transport service; and (3) the urban mobility instruments, as SUMP in its diagnosis, monitoring and evaluation phases, both at municipal and metropolitan levels [4,6,7,8,60]. 

## 7. Conclusions

Urban mobility is clearly related to urban livability, but the debate about its measurement is still ongoing. Considering the analysis of the already existing indicators in Portugal, some conclusions can be made. The factors generated by a principal component analysis (PCA) are clearly related with the conceptual discussion that links urban livability and urban mobility, demonstrating some domain diversity (e.g., commuting, motorization, environment, civics, and health), despite the reduced number of available indicators. The cluster analysis based on PCA values distinguished intrametropolitan differences, since it groups municipalities with similar profiles of mobility patterns and problems. The results show a strong relation with the urban expansion process, urban densities, and distance to Lisbon, which generates diverse types of urban occupation, different dependency levels to Lisbon, namely due to employment, and, consequently, different mobility patterns.

The identification of such differences, highlighting sensitive territories from the perspective of urban livability premises, is one of the first tasks for an effective urban planning. As transportation has over time allowed people to multiply their living places beyond the area of residence, especially in metropolitan areas where there are strong networks of interdependence between territories, it becomes important to have not only a local approach to livable communities but also an integrated territorial approach, on a metropolitan scale, oriented toward a livable metropolis.

This study points out the need to strengthen statistics systems from a multilevel approach, reinforcing the work between national statistics producing entities, municipal entities, and companies with intervention in the area, in order to generate new data from geographic information systems or real-time collection data tools, and/or include data about recent domains, such as the number of users and types of use of vehicle sharing platforms; the size and use of cycle paths; the use of teleworking, telecommuting, and teleservices to replace physical travel; and the number of modernized public collective transport vehicles. This will certainly reinforce the understanding of the debate of the contribution of urban mobility to livable communities and livable metropolises.

## Figures and Tables

**Table 1:** Domains/indicators of transport and urban mobility for measuring urban livability.

Main Domains	No. of References	Examples
Active transport and public transport	18	Bicycle lanes and footpaths (availability, quality, ability to ride a bike) Public transport availability, length, quality, capacity, number of stops/stations, number of vehicles, satisfaction, access
Transport infrastructure	13	Road infrastructure: length, length per capita, density, access, quality level; Network average speed, road traffic, congestion
Accessibility	13	Distance/time: average distance to equipment, goods, or services and to city center Distance/time: average distance to bus stops, railway stations, subway stations
Road safety	12	Traffic accidents, deaths from traffic accidents, economic loss per traffic accident, road signs, protection of street with priority for pedestrians, safe sidewalks and overpasses, separation of pedestrians and road traffic
Environment	8	Air pollution: CO_2_ emissions, concentrations of PM2.5 and PM10, days with good air quality Noise pollution from traffic
Economy and energy	5	Expenditure on transportation per capita, transportation costs Energy consumption
Others	3	Parking

Source: Adapted from Khorrami, Z., et al [51].

**Table 2:** Indicators from official entities and respective metadata. INE, National Institute of Statistics; CRA, Vehicle Registry Office; ANSR, National Road Safety Authority; DGPJ, Directorate-General for Justice Policy; PROT-AML, Regional Land Use Management Plan of the Lisbon Metropolitan Area; APA, Portuguese Agency for Environment; DGEG, Directorate-General for Energy and Geology.

	Indicator	Unit	Year	Source
Land occupancy	(1) Population density	%	2011	INE
**Axis 1: Urban mobility**				
Domain 1 Commuting: Main destination	(2) Local commuting (travel to residence parish)	%	2011	INE
	(3) Intermunicipal commuting (travel to another municipality)	%	2011	INE
	(4) Population entering municipality for daily work	%	2011	INE
Domain 2 Commuting: Main transport mode	(5) Commuting by car	%	2011	INE
	(6) Commuting by public transport	%	2011	INE
	(7) Commuting on foot	%	2011	INE
Domain 3 Commuting: Travel time (one trip)	(8) Short commute (up to 15 min)	%	2011	INE
	(9) Long commute (longer than 60 min)	%	2011	INE
Domain 4 Motorization	(10) New light passenger vehicles sold per 1000 inhab.	No./1000 inhab.	2016	INE
	(11) Motorization rate (light vehicles)	No./1000 inhab.	2016	CRA
**Axis 2: Mobility, public health, and civics**				
Domain 5 Road accidents	(12) Traffic accident victims per 1000 inhab.	Victims of road accidents/1000 inhab.	2016	ANSR/INE
	(13) Injury severity index of road accidents with victims	Killed on road accidents/road accidents × 100	2016	ANSR/INE
Domain 6 Transport-related crime	(14) Crime rate for driving under the influence of alcohol (alcohol level ≥ 1.2 g/L)	%	2017	DGPJ/INE
	(15) Crime rate for driving without legal qualification	%	2017	DGPJ/INE
**Axis 3: Mobility, public health, and environment**				
Domain 7 Carbon emissions	(16) Carbon emissions from transportation sector per 1000 inhab.	Ton CO_2_/1000 inhab.	2011	PROT-AML/APA
Domain 8 Fuel	(17) Car fuel consumption per inhabitant	tonne of oil equivalent (toe) inhab.	2016	DGEG/INE

**Table 3:** Loadings and component eigenvalues.

		Variables	Components				
			1	2	3	4	5
		(1) Population density	**−0.822**	0.347	−0.074	−0.083	0.032
Axis 1: Urban transport and mobility	Domain 1 Commuting, main destination	(2) Local commuting (travel to residence parish)	**0.921**	−0.107	0.147	0.011	0.069
		(3) Intermunicipal commuting (travel to other municipality)	−0.226	**−0.843**	0.033	0.141	0.100
		(4) Population entering municipality for daily work	−0.253	**0.893**	−0.002	0.165	0.188
	Domain 2 Commuting, main transport mode	(5) Commuting by car	**0.682**	0.054	**0.708**	−0.027	0.096
		(6) Commuting by public transport	**−0.937**	0.005	−0.048	−0.090	0.148
		(7) Commuting on foot	0.006	0.007	**−0.935**	−0.011	−0.016
	Domain 3 Commuting, time spent (one trip)	(8) Short commute (up to 15 min)	**0.949**	0.111	−0.094	−0.003	0.137
		(9) Long commute (longer than 60 min)	0.169	−0.434	**−0.579**	0.070	**−0.571**
	Domain 4 Motorization	(10) New light passenger vehicles sold per 1000 inhab.	−0.352	**0.752**	0.254	0.028	−0.030
		(11) Motorization rate (light vehicles)	0.072	**0.507**	**0.749**	0.019	−0.189
Axis 2: Transport, public health, and civics	Domain 5 Road safety	(12) Traffic accident victims per 1000 inhab.	0.014	**0.789**	0.364	0.371	0.215
		(13) Injury severity index of road accidents with victims	**0.600**	−0.197	−0.198	0.175	**0.632**
	Domain 6 Transport-related crime	(14) Crime rate for driving under the influence of alcohol (alcohol level ≥ 1.2 g/L)	−0.003	**0.749**	0.056	0.497	−0.199
		(15) Crime rate for driving without legal qualification	0.029	0.309	−0.333	**0.804**	−0.251
Axis 3: Transport, public health and environment	Domain 7 Carbon emissions	(16) Carbon emissions from transportation sector per 1000 inhab.	0.122	−0.017	0.240	**0.835**	0.234
	Domain 8 Fuel	(17) Car fuel consumption per inhabitant	**0.545**	0.302	−0.264	0.448	0.269
	Eigenvalue		5.067	4.76	2.591	1.307	1.019
	% of variance		29.806	27.999	15.240	7.690	5.997
	Cumulative % of variance		29.806	57.805	73.045	80.735	86.732

Factor loadings superior to 0.500 highlighted in bold.

## Data Availability

Publicly available datasets were analyzed in this study. This data can be found here: https://www.ine.pt/xportal/xmain?xpgid=ine_main&xpid=INE&xlang=pt; https://apambiente.pt/ (accessed on 29 March 2021).

## Appendix A

**Table A1:** Cluster scores and socioeconomic variables by PCA component.

Clusters	Components					Pop. Density 2011 (Inhab./km^2^)	Average Distance to Lisbon Major Urban Centers by Car (km)	Employment Attraction Rate (% of Residents)
	C1	C2	C3	C4	C5			
A	−0.81	−0.53	0.00	−0.11	0.37	2715	22.4	11.9
B	0.01	−1.03	−1.28	−0.73	−0.91	1221	41.0	6.7
C	1.22	0.37	−0.25	−0.52	−0.30	266	42.7	12.2
D	0.33	0.17	1.51	−0.49	−0.63	1824	31.7	19.8
E	0.03	−0.27	−2.13	1.24	−1.97	2171	37.0	12.9
F	1.48	0.00	−0.87	0.39	2.78	101	41.0	24.3
G	0.61	−0.04	1.02	3.10	0.05	115	47.0	24.0
H	−1.12	3.44	−0.50	−0.17	0.26	6645	0.0	77.7

**Table A2:** Pearson’s correlation between cluster scores and socioeconomic variables by PCA component.

	C1	C2	C3	C4	C5
Pop. density 2011 (inhab./km^2^)	−0.85746	0.779223	−0.11731	−0.27728	−0.14123
Average distance between Lisbon and major urban centers by car (km)	−0.89592	0.85031	−0.13634	−0.26102	−0.16353
Employment attraction rate (% of residents)	−0.93532	0.899425	−0.17987	−0.32285	−0.19551
